# Nickel (II) and Cobalt (II) Alginate Biopolymers as a “Carry and Release” Platform for Polyhistidine-Tagged Proteins

**DOI:** 10.3390/gels8020066

**Published:** 2022-01-18

**Authors:** Andrei-Mihai Dumitrașcu, Iuliana Caraș, Cătălin Țucureanu, Andreea-Laura Ermeneanu, Vlad-Constantin Tofan

**Affiliations:** Cantacuzino National Military Medical Institute for Research and Development, 050096 Bucharest, Romania; dumitrascu.andrei@cantacuzino.ro (A.-M.D.); caras.iuliana@cantacuzino.ro (I.C.); tucureanu.catalin@cantacuzino.ro (C.Ț.); andreea.ermeneanu@gmail.com (A.-L.E.)

**Keywords:** nickel alginate, cobalt alginate, polyhistidine-tagged proteins, biopolymer, hydrogel

## Abstract

Protein immobilization using biopolymer scaffolds generally involves undesired protein loss of function due to denaturation, steric hindrance or improper orientation. Moreover, most methods for protein immobilization require expensive reagents and laborious procedures. This work presents the synthesis and proof of concept application of two alginate hydrogels that are able to bind proteins with polyhistidine tags by means of interaction with the crosslinking cations. Nickel (II) and cobalt (II) alginate hydrogels were prepared using a simple ionic gelation method. Hydrogels were characterized by optical microscopy and AFM, and evaluated for potential cytotoxicity. In addition, binding capacity was tested towards proteins with or without HisTAG. Hydrogels had moderate cytotoxicity and were able to exclusively bind polyhistidine-tagged proteins with a binding capacity of approximately 300 µg EGFP (enhanced green fluorescent protein) per 1 mL of hydrogel. A lyophilized hydrogel-protein complex dissolved upon the addition of PBS and allowed the protein release and regain of biological activity. In conclusion, the nickel (II) and cobalt (II) alginate biopolymers provided an excellent platform for the “carry and release” of polyhistidine-tagged proteins.

## 1. Introduction

There is an ever-growing need for easy, safe and reliable drug or protein delivery systems, so the world is focusing its attention, more than ever, towards natural biopolymers [1]. Therefore, even though they have been used for a long time in microbiology under the form of agar gels [2], or in electrophoresis [3] and protein purification techniques as agarose gels [4], biopolymers such as agar, gelatin, chitosan and alginate are still studied to generate new applications in the form of food additives, wound dressings or cell scaffolds [5,6,7,8]. Yet, when there is talk about using them in vivo, one has to take into account the fact that these polymers need to be biocompatible, and they need to keep the bound molecules in their active form.

Immobilization of proteins using biopolymers as a scaffold often requires normal covalent binding; thus, modifying particular aminoacids residues that might affect stability or biological activity [9,10,11,12]. Moreover, normal covalent binding is usually targeted towards more than one residue per protein molecule generating multiple possible orientation that can affect substrate availability (enzymes) or epitope recognition by antibodies or T-cell receptors (TCR) [13]. All these disadvantages are accompanied by high costs and varying coupling turnout.

Non-covalent immobilization methods of proteins are not exotic either, but they generally imply the incorporation of protein molecules in bulk biopolymers. This strategy also obstructs protein availability and can induce protein denaturation [14,15,16,17].

One example of a successful non-incorporating large-scale use strategy for biopolymer protein immobilization is used in a type of affinity chromatography termed IMAC (immobilized metal affinity chromatography) [18,19]. IMAC uses agarose beads with grafted chelating moieties (iminodiacetic acid—IDA, nitrilotriacetic acid—NTA). IDA or NTA can fix certain divalent cations (Ni^2+^, Co^2+^, Cu^2+^, etc.) that, in turn, are able to use two or three of their remaining coordinate bonds in binding the imidazole ring of histidine, a common aminoacid. If a protein contains four or more consecutive histidines placed at one end of its primary sequence, it can bind to the divalent cation-coated agarose bead. As sequences of four or more histidines are not a common natural trait, recombinant technologies use gene modifications to insert these sequences. These sequences are called HisTAG, and their primary use is assisting in purifying recombinant proteins through IMAC [20,21,22].

Yet, the use of IMAC technology for protein storage or delivery is hindered by several impediments. First, protein release/elution is usually accomplished by adding high amounts of imidazole or highly acidic buffers [23], which can limit its use in biological systems. Additionally, agarose is virtually insoluble at room temperature in aqueous solutions, so that its use for in vivo applications might require an additional agarose removal step [24].

Sodium alginate is one of the most researched natural polymers to date. It is derived from the alginic acid, a natural polysaccharide extracted from brown algae cell walls or found in bacteria. This linear copolymer is formed from linked 1–4 β-d-mannuronic acid and α-l-guluronic acid block residues in either a homogeneous or heterogeneous conformation (-MMMM-/-GGGG-; MGMGMGMG-). This conformation, together with the G:M ratio and its molecular mass, can influence alginate viscosity or fragility [18].

When sodium alginate is combined with divalent metallic ions (Ca^2+^, Zn^2+^, Ba^2+^, Cd^2+^, etc.), it forms a type of gel by replacing the sodium ions with said cations. During this exchange, the polymeric chains will form an “egg-box” network with the metallic ions as their nodes [25,26]. Moreover, the process is fully reversible as ionic crosslinked alginate gels lose their structure upon the addition of sodium-containing solutions, such as PBS (phosphate buffer saline) or saline solution. Applications of ionic crosslinked alginates range from drug delivery, scaffolds for tissue engineering, 3D printing [27,28], antibiotics removal [29], and even improving the flame retardancy of some materials [30].

Previous work on mimicking IMAC using alginate was carried by Dalal et al. [31]. In this instance, authors used chemically crosslinked alginate that was afterwards charged with divalent cations, like Ni^2+^. They claim that this version of crosslinked alginate can aid in the purification of proteins, even non-HisTAG proteins.

In this study, we propose the synthesis of alginate hydrogels based on the crosslinking of alginate with nickel (II) or cobalt (II) that can mimic IMAC preferential protein binding and, moreover, are fully solvable in vivo or in common buffers due to lack of chemical crosslinking. Hydrogels are produced using a simple, straightforward ionic gelation method. Moreover, the Ni^2+^ and Co^2+^ alginates are able to preferentially bind his-tagged proteins in hydrogel form, retain them after lyophilization and, upon the addition of physiological solutions, release them without affecting biological activity, together with dissolution of the scaffold. Features of these hydrogels might make them useful in the biomedical field, acting as vaccine carriers, and as biocompatible injectable scaffolds that can immobilize proteins of interests while preserving their biological activity.

## 2. Results and Discussion

Nickel (II) and cobalt (II) alginates were prepared using an ionic gelation method [32] employing the ultrasonic dispersion of sodium alginate into a NiCl_2_ or CoCl_2_ solution under continuous stirring [33]. The result consisted of a solution with a heterogeneous appearance, with scattered fine aggregates throughout the entire volume. After excess crosslinker removal by repeated centrifugation and deionized water washings, a viscous quasi-homogeneous hydrogel was formed. This hydrogel could flow and could be easily manipulated with a serological pipette.

Efforts to replicate alginate hydrogel formation with other divalent cations using the same method were made. Ca^2+^, the most common alginate crosslinking agent, formed multiple solid aggregates that would not coalesce into the same type of viscous hydrogel. Moreover, previous experiments showed that calcium alginate beads bind his-tagged proteins poorly (Figure S1, in the Supplementary Materials), as compared to Ni^2+^ and Co^2+^ alginate beads. Cu^2+^, a decent candidate for his-tagged protein binding, formed a rather finely dispersed mix of solid aggregates that would sediment in a short period of time.

### 2.1. Characterization of Alginate Hydrogels

**Figure 1:**
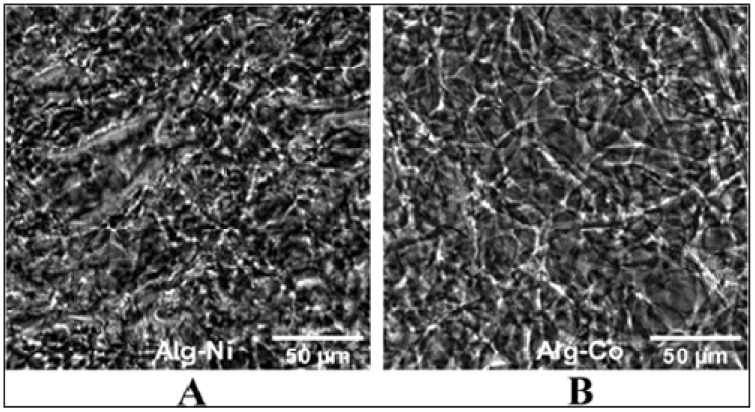
Phase contrast images of nickel (II) alginate (**A**) and cobalt (II) alginate (**B**) -dried hydrogels. Scale bar represents 50 µm.

In order to characterize the synthesized hydrogels, both microscale and nanoscale observations were made. Phase contrast micrographs were recorded on oven-dried samples, as it was previously observed, they adequately preserved the hydrogel microarchitecture. Figure 1 shows an intricate network of spindle-like structures for each of the dried hydrogel with a slightly more organized pattern in the case of cobalt (II) alginate.

**Figure 2:**
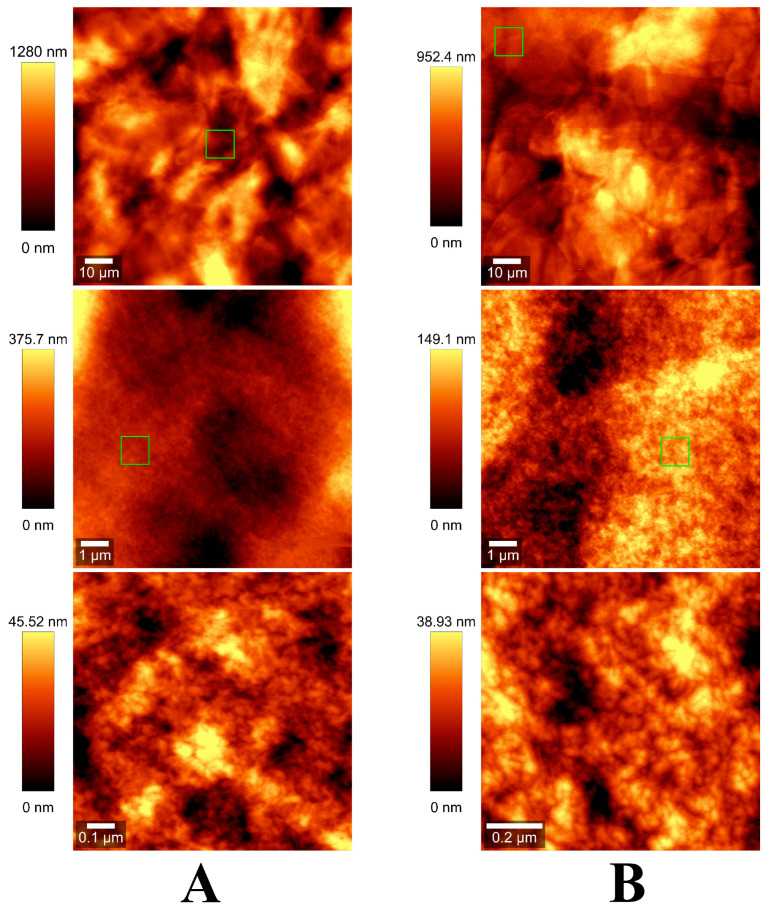
AFM surface topography scan of nickel (II) alginate (**A**) and cobalt (II) alginate (**B**). From top to bottom: 100 µm × 100 µm, 10 µm × 10 µm and 1 µm × 1 µm scans. Green square represents an area scanned at a higher resolution.

Roughly the same observations can be made from evaluating a coarse (100 µm × 100 µm) AFM surface topography scan of the same dried samples (Figure 2 and Figure S2). Yet, when a more in-depth scan was performed, features with uniform size distribution of about 10–20 nm were discernible (Figure S3, in the Supplementary Materials). These observations hinted towards the hypothesis that nanodrops are formed when dispersing alginate drops with ultrasonic waves. Upon reaching the crosslinking solution, these alginate nanodrops underwent rapid cation exchange and formed nanometric nickel/cobalt alginate structural motifs. Subsequently, due to both crowding and crosslinker excess, these nanostructures assembled in higher order macroscopic structures. This might also have been enhanced by the stacking due to centrifugation. Following the removal of the unbound cations in the washing steps, intermolecular electrostatic interactions might occur leading to the formation of the hydrogel.

### 2.2. Cytotoxicity Assessment of Alginate Hydrogels

When designing or modifying a polymeric material with intended in vivo use, the matter of its biocompatibility should be of primary concern. Alginates, at least in their most common form as sodium or calcium salts, are widely considered to be biocompatible. Likewise, more often than not, scientific focus is directed on calcium alginate due to calcium’s presence in physiological fluids leading to their assumed lack of toxicity [34].

Nickel is not considered an essential nutrient in humans, but it is important for human health as it assists the bacteria in microbiota. Its toxicity is usually related to overexposure (occupational exposure, oral ingestion) and direct skin contact that leads, in some cases, to nickel allergic contact dermatitis [35,36]. Cobalt is an essential nutrient, being a constituent of vitamin B_12_ or cobalamin, but it can also cause health complications and skin allergies [37].

In order to evaluate potential toxicity, both alginates were tested for cytotoxic effects in vitro, using two murine cell lines, L929 and RAW264.7. L929 fibroblast cell line was used on the grounds that it is the golden standard for testing cytotoxic effects [38] and, considering that allergic reactions have an immune component, RAW264.7 macrophage-like cell line was used to test toxicity towards the immune cells [39].

**Figure 3:**
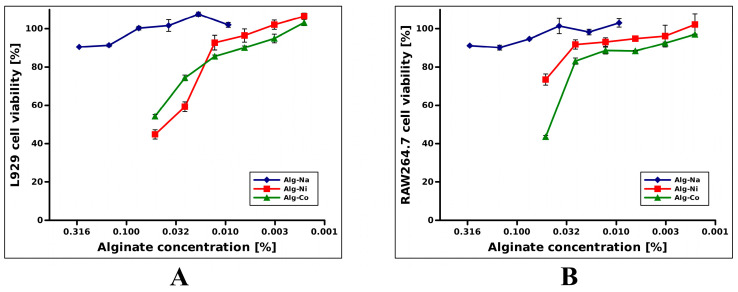
Cytotoxicity evaluation of nickel (II) and cobalt (II) alginates on different cell lines. (**A**) L929 cell viability; (**B**) RAW264.7 cell viability. Cell viability values were normalized considering positive control (cells cultured in DMEM alone) as having 100% viability. All samples were in triplicate. Values are represented as mean ±+/− SEM. X axis is log transformed.

Figure 3 shows the effect of tested alginates on cell viability. For easier comparison with sodium alginate, concentrations of tested samples were adjusted to total alginate content (considering 100% alginate reticulation on synthesis, Table S1). In our experimental setting, L929 cells were affected by higher concentrations of nickel (II) or cobalt (II) alginates. Viability of above the 80% threshold resulted in concentration values of up to 0.01% (Figure 3A). Similar results were obtained for RAW264.7 cell viability, as well (Figure 3B). Additionally, an 80% viability threshold was achieved at concentration values of 0.025%. Nonetheless, cytotoxicity of tested alginates was lower than NiCl_2_ and CoCl_2_ at the same concentration (Figure S4)). Therefore, it is safe to assume that hydrogel toxicity was related to their Ni^2+^ or Co^2+^ content.

**Figure 4:**
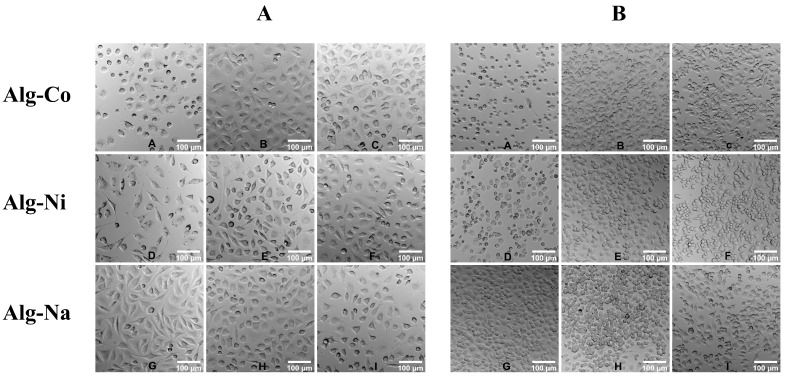
Phase contrast images of L929 (**A**) and RAW264.7 (**B**) cells grown in the presence of Alg-Co (cobalt alginate), Alg-Ni (nickel alginate) and Alg-Na (sodium alginate). Alg-Co—A = 0.05%, B = 0.01%, C = 0.001%; Alg-Ni—D = 0.05%, E = 0.01%, F = 0.001%; Alg-Na—G = 0.3%, H = 0.075%, I = 0.001.

Cells were also examined microscopically when cultured in the presence of different alginate concentrations. Normal L929 cell morphology is spindle-like, as it can be observed in the case of cells cultured with sodium alginate (Figure 4A, Alg-Na). High concentrations of Co^2+^ and Ni^2+^ alginates resulted in lower cell density and cells with a rounder morphology, a clear sign of cytotoxicity (Figure 4A). Moreover, RAW264.7 cells had a flat, spread morphology when cultured with sodium alginate, as opposed to fewer and round cells that were observed for the other two conditions (Figure 4B).

Although use of hydrogels might be restricted to lower concentrations due to cytotoxicity, their potential intended use as protein carriers/scaffolds allows for the utilization of low amounts that are below the cytotoxicity limit.

### 2.3. Alginate Hydrogels Specific Binding of Proteins

To test if alginate gels can bind his-tagged proteins, three fluorescent proteins were used, EGFP (enhanced green fluorescent protein), EGFP-HisTAG and TurboRFP (red fluorescent protein). These proteins were deemed adequate due to their stable fluorescence and their ability to also emit on UV excitation [40].

**Figure 5:**
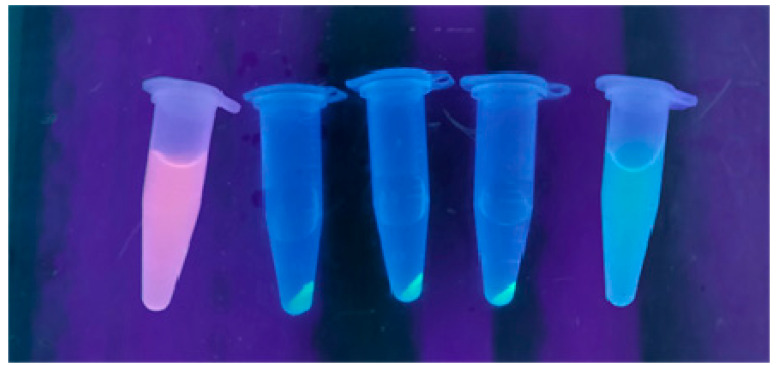
Assessment of preferential binding of his-tagged proteins to Ni^2+^ and Co^2+^ alginate hydrogels. Samples incubated with different fluorescent proteins were centrifuged and UV illuminated. From right to left: Ni^2+^ alginate + TurboRFP; Ni^2+^ alginate + EGFP-HisTAG; Co^2+^ alginate + EGFP-HisTAG; Ni^2+^ alginate + EGFP-HisTAG; Ni^2+^ alginate + EGFP.

Both hydrogels were mixed with 12.5 µg of each protein and, after incubation at room temperature, the suspensions were centrifuged. The results showed that both nickel (II) and cobalt (II) alginates bound EGFP-HisTAG as the sedimented hydrogel-emitted fluorescence, while the rest of the solution did not. Moreover, an identical protein (EGFP), but lacking HisTAG, was uniformly dispersed in the solution, indicating a lack of binding. Finally, the lack of binding was also observed when incubated with TurboRFP (Figure 5). These results suggest that the hydrogels do not bind proteins, through adsorption or electrostatic forces, but preferentially bind his-tagged proteins.

Next, binding capacity of hydrogels was quantified. Two incubation times were tested. Table 1 shows the percentage of nickel (II) alginate hydrogel-immobilized EGFP-HisTAG when different amounts were added. Cobalt (II) alginate hydrogel performed comparably (Table 2).

The results indicated that over 90% of the total protein was bound when incubation with 37.5 µg protein lasted 1 h, or when incubation with 12.5 µg protein lasted 24 h, in the case of nickel (II) alginate. This amounts to approximately 300 µg protein/mL hydrogel for 1 h and, if referring to dry mass, this corresponds to 20 mg protein/g dried hydrogel. For comparison, Ni-NTA agarose beads can bind up to 60 mg/mL gel, which means up to 200× more than nickel (II) alginate hydrogel. In spite of much lower binding capacity, the results are still encouraging, as most biomedical applications (vaccines, enzymes, immunostimulants) require quality protein in the range of micrograms [41].

One would expect that longer incubation times would improve protein binding. In this case, the opposite is valid, as binding decreases over time. This is probably due to nickel leakage driven by the protein itself, as multiple molecules compete for the same coordination bonds. Moreover, normal nickel leakage might also take place as the equilibrium might slowly shift over time towards free Ni^2+^ in solution.

As longer incubation times correlate to the binding of lower protein amounts, the prospect of lyophilization of hydrogels incubated with EGFP-HisTAG was explored (Figure S5). This way, due to lack of an aqueous environment, protein mobility would be restricted, leading to no competition for nickel coordination bonds. Moreover, lack of water would prohibit Ni^2+^ normal leakage. However, due to dehydration, immobilized proteins might lose their biological activity.

**Figure 6:**
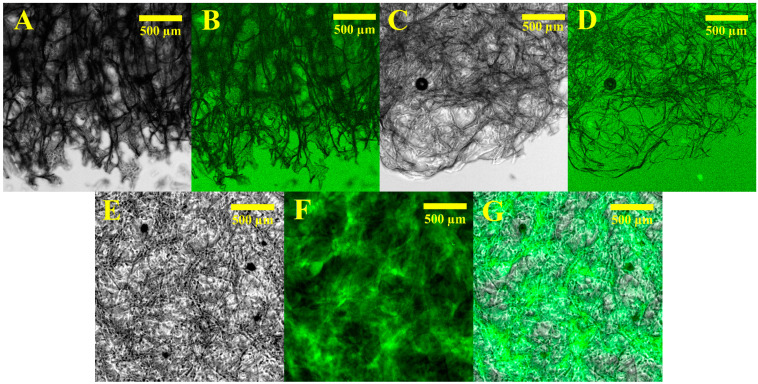
Phase contrast (**A**,**C**,**E**) and fluorescence (GFP) (**B**,**D**,**F**) images of lyophilized nickel (II) alginate samples without (**A**,**B**) and with (**C**,**D**) bound EGFP-HisTAG. Second row (**E**,**F**) shows a rehydrated sample with immobilized EGFP-HisTAG. (**G**) overlap of (**E**,**F**).

In order to rule out the loss of biological activity upon loss of water, lyophilized samples and rehydrated samples were analyzed for fluorescence activity specific to EGFP-HisTAG. Lyophilized samples with or without protein were observed in both phase contrast and GFP fluorescence (Figure 6A–D). Both samples appeared similar, with no fluorescent signal, suggesting no EGFP fluorescent activity in the dried state.

However, on rehydration with PBS (phosphate buffer saline) of the EGFP-HisTAG containing the nickel (II) alginate sample, the lyophilized sample lost its integrity and released the fluorescent protein in the aqueous environment (Figure 6E–G). Sodium and potassium ions in PBS were found in great excess, and replaced the nickel in the alginate structure. At the same time, EGFP was released and regained its fluorescence.

## 3. Conclusions

Nickel (II) and cobalt (II) alginate hydrogels were synthetized using an ionic gelation method. Macroscopic examination of the two hydrogels revealed an intricate network of packed plate-like structures. On in-depth evaluation, 10 nm regular features were discernible leading to a nanostructure aggregation hypothesis for hydrogel formation.

Hydrogels displayed moderate cytotoxicity towards L929 and RAW264.7 at highest tested concentrations due to their Ni^2+^ and Co^2+^ content, but exhibited little to none at working dilutions.

Hydrogels were shown to exclusively bind polyhistidine-tagged proteins with a binding capacity that was suitable for biomedical applications. Moreover, lyophilized hydrogels with immobilized EGFP-HisTAG dissolved upon the addition of PBS and allowed the regain of biological activity and the release of EGFP-HisTAG.

## 4. Materials and Methods

### 4.1. Materials

Sodium alginate (medium viscosity, high G/M ratio, cat. no. 71238), nickel (II) chloride hexahydrate, cobalt (II) chloride hexahydrate were purchased from Sigma. Enhanced green fluorescent protein (EGFP), turbo red fluorescent protein (TurboRFP) and their HisTAG varieties were previously synthesized in-house using bacterial recombinant expression. Briefly, plasmidial vectors containing the genes for the fluorescent proteins were transformed into BL21(DE3) *E. coli* strain. Bacteria were cultured in 2YT culture medium until reaching an optical density value of 0.8. 1 mM IPTG (isopropyl β-d-1-thiogalactopyranoside) was added to the culture and after 3 h bacteria were harvested by centrifugation. Cold osmotic shock [30] was used to separate soluble protein. Afterwards, ion-exchange chromatography and/or affinity chromatography was used for purification.

L929 and RAW264.7 cell lines were purchased from ECACC. DMEM (Dulbecco’s modified eagle medium) cell culture medium, l-glutamine, penicillin/streptomycin and fetal bovine serum (FBS) were acquired from Lonza (Guangzhou, China), Sigma (Burlington, MA, USA) or Biochrom (Waterbeach, UK).

### 4.2. Alginate Hydrogel Synthesis

A 0.3 % *w*/*v* aqueous sodium alginate solution was delivered with a 0.25 mL/min flow rate in the proximity of a ⌀ 3 mm titanium sonotrode tip attached to a Labsonic M (Braun Biotech International, Stockholm, Sweden) operated at A = 100% and ν = 0.6 s^−1^. The alginate solution was dispersed in a 0.3% *w*/*v* aqueous M (II) chloride solution (M = Ni, Co) under continuous stirring. Final volumetric ratio was 3:25 (alginate solution:divalent cation chloride solution). After complete mixing, the solution containing crosslinked alginate was subjected to repeated wash cycles, consisting of centrifugation for 3 min at 4000× *g* and dispersion in deionized water for the removal of excess divalent cation.

Final alginate hydrogel volume was 0.58× of alginate solution prior to crosslinking. Hydrogels were stored at 4 °C until further use. Lyophilized samples were prepared as described in [42], using an Alpha 1–4 LSC equipment at a temperature of −70 °C, pressure of 0.055 mBar for 72 h.

### 4.3. Cell Culture and Cytotoxicity Assay

L929 murine fibroblast cells and RAW264.7 murine macrophage cells were cultured in complete DME medium (DME supplemented with 5% FBS, 100 U/mL antibiotics and 2 mM l-glutamine) in cell culture flasks incubated at 37 °C, humidified atmosphere and 5% CO_2_. Cells were enzymatically detached and seeded in 96-well culture plates at a cell density of 1 × 10^4^ cells/well (L929) or 2.5 × 10^4^ cells/well (RAW264.7). After cell attachment, the supernatant was discarded and replaced with serial dilutions of synthesized hydrogels. Sodium alginate was also tested for comparison. Positive control was represented by cells cultured in complete cell culture medium, whereas the negative control consisted of cells exposed to 0.3% *w*/*v* NiCl_2_ or CoCl_2_. After overnight incubation, the supernatant was discarded and replaced with DMEM containing 0.5 mg/mL MTT (3-[4,5-dimethylthiazol-2-yl]−2,5-diphenyltetrazolium bromide). After another 3 h incubation, lysis solution (20% *w*/*v* sodium dodecyl sulfate, 50% *w*/*v* N,N-dimethylformamide, 0.4% *w*/*v* acetic acid, 0.04 M hydrochloric acid) was added and sample absorbance was recorded at 560 nm.

Cell morphology and cell density was observed using a Nikon eclipse Ti inverted microscope using a 10×/0.30 objective.

### 4.4. Specific His-Tagged Protein Binding Assessment

Equal amounts of EGFP, TurboRFP and EGFP-HisTAG were mixed with 1/16 aqueous dilution of alginate hydrogels and incubated for 1 h at room temperature. Samples were centrifuged for 60 min at 21,000× *g* and fluorescence signal was observed on a UV transilluminator.

### 4.5. Protein Binding Capacity Assay

Nickel (II) alginate hydrogels were diluted 1/16 with deionized water and incubated with different EGFP-HisTAG amounts in low-binding microcentrifuge tubes for 1 h and 24 h at room temperature. After incubation, samples were centrifuged for 60 min at 21,000× *g* and the supernatant was collected. Supernatant fluorescence was measured using a Tecan Infinite M1000 microplate reader using excitation wavelength of 488 nm and emission wavelength of 515 nm. Values were examined against a standard curve plotted from fluorescence values of EGFP-HisTAG in hydrogel-free solutions. The amount of immobilized protein was calculated as difference between total protein and protein in supernatant (non-bound).

### 4.6. Optical and Fluorescence Imaging Investigation of Dried or Lyophilized Hydrogels

Phase contrast images were taken using a Nikon DS-Qi2 camera connected to a Nikon eclipse Ti inverted microscope and a 4×/0.13 objective. Fluorescence images were generated using UV illumination and a GFPQ filter (EX: 455–485 nm, DM: 495 nm, EM: 500–545 nm). Micromanager (v. 1.4.23) and Fiji ImageJ (v. 1.53c) software products were used to capture and edit the images.

For dried samples, 50 µL of each hydrogel on a microscope glass slide was dried at 110 °C for 1 h. For lyophilized samples, Alg-Ni samples with or without EGFP-HisTAG were fixed between glass slides and analyzed in non-hydrated and hydrated states.

### 4.7. AFM Surface Characterization

Atomic force microscopy scans were made in amplitude modulation mode using a Witec alpha300RAS+ equipment and 240AC-NG cantilevers with resonance frequency = 70 kHz and force constant = 2 N/m (Opus). Phase and topography scans were recorded from oven dried samples on 100 µm × 100 µm, 10 µm × 10 µm and 1 µm × 1 µm surfaces.

## Figures and Tables

**Table 1:** Nickel (II) alginate binding capacity of EGFP-HisTAG over two incubation times.

EGFP-HisTAG (µg)	Incubation Time (h)	
	t_1_ = 1 h	t_2_ = 24 h
12.5 µg	98.45%	95.05%
25 µg	97.15%	87.01%
37.5 µg	93.52%	67.16%
50 µg	87.28%	56.11%
62.5 µg	73.56%	43.14%

**Table 2:** Cobalt (II) alginate binding capacity of EGFP-HisTAG over two incubation times.

EGFP-HisTAG (µg)	Incubation Time (h)	
	t_1_ = 1 h	t_2_ = 24 h
12.5 µg	96.87%	78.50%
25 µg	85.91%	67.50%
37.5 µg	77.24%	59.77%
50 µg	71.92%	57.44%
62.5 µg	66.40%	52.35%

## Supplementary Materials

The following are available online at https://www.mdpi.com/article/10.3390/gels8020066/s1, Figure S1: Overlapped phase and fluorescence microscopy images of alginate beads, Figure S2: AFM surface phase scan of nickel (II) alginate—A and cobalt (II) alginate—B., Figure S3: AFM surface phase scan crossection for nickel (II) alginate—A and cobalt (II) alginate—B, Table S1: Gravimetric analysis of calcinated nickel (II) alginate, Figure S4: Cytotoxicity evaluation of nickel (II) and cobalt (II) alginates on different cell lines, Figure S5: Lyophilized nickel (II) alginate sample with immobilized EGFP-HisTAG.
