# Supplementary material for: Nickel (II) and Cobalt (II) Alginate Biopolymers as a “Carry and Release” Platform for Polyhistidine-Tagged Proteins
Source: Gels. 2022 Jan 18;8(2):66. doi: 10.3390/gels8020066 (PMC8871198; doi:10.3390/gels8020066)
Supplement: Supplementary file 1 [file gels-08-00066-s001.zip › gels-1510859-supplementary.pdf]

Nickel (II) and cobalt (II) alginate biopolymers as a "carry and release" platform for polyhistidine-tagged proteins

Andrei-Mihai Dumitrașcu [1], Iuliana Caraș [1], Cătălin Țucureanu [1], Andreea-Laura Ermeneanu [1] and Vlad-Constantin Tofan [1,*]

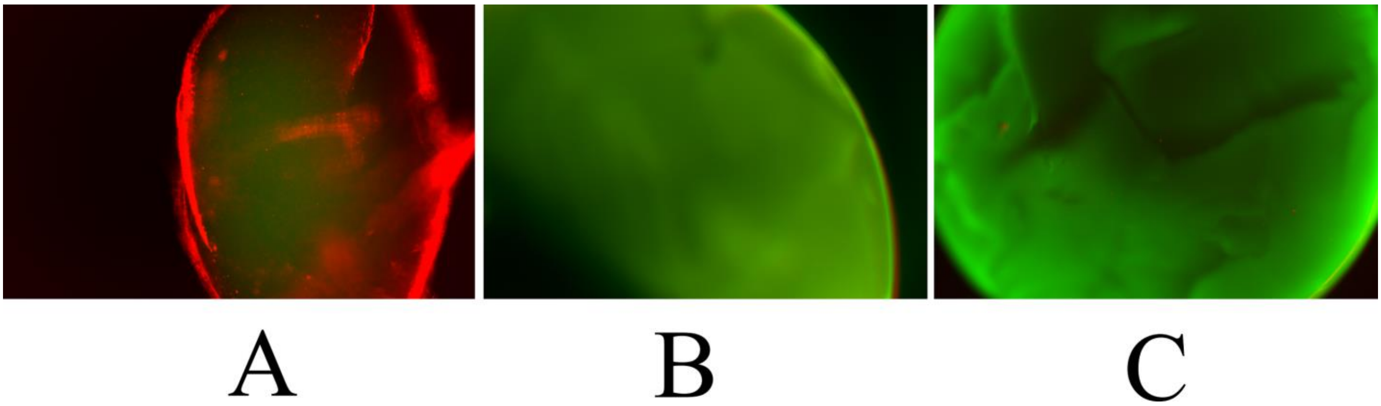

Figure S1. Overlapped phase and fluorescence microscopy images of alginate beads. Beads were obtained using an internal gelation method from 5% w/w $CaCl_2$, $CoCl_2$ or $NiCl_2$ and 2% sodium alginate. A – calcium alginate; B – cobalt alginate; C – nickel alginate.

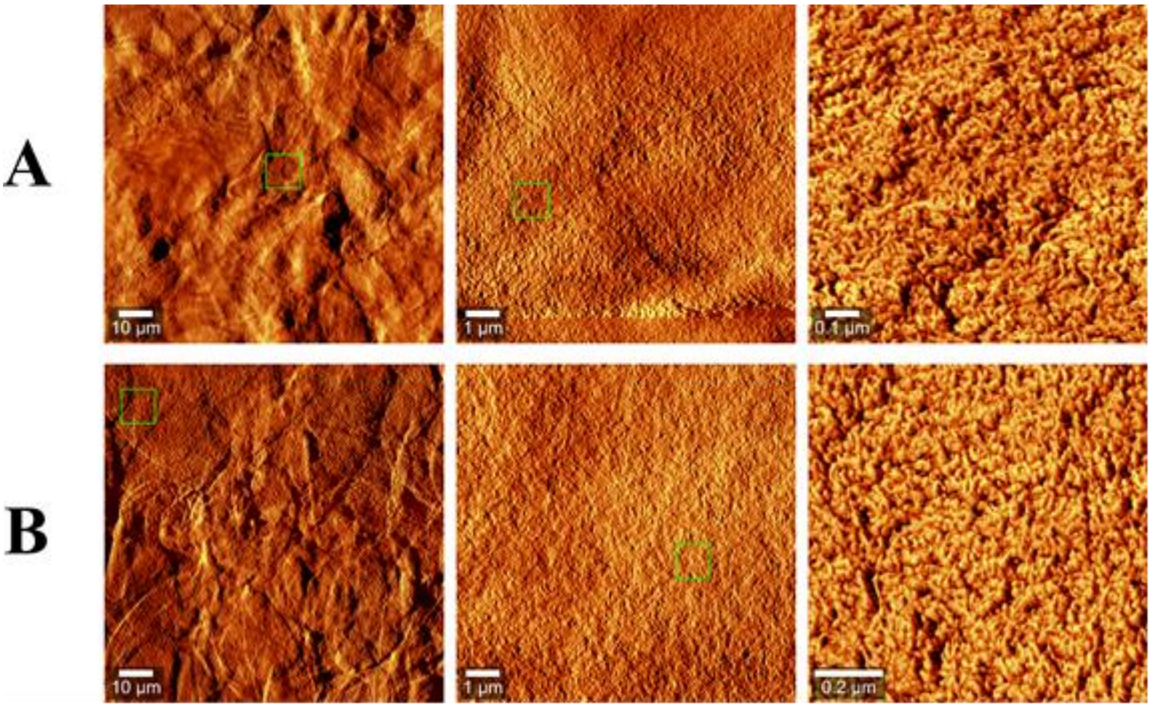

Figure S2. AFM surface phase scan of nickel (II) alginate – A and cobalt (II) alginate – B. From left to right: 100 µmx100 µm, 10 µmx10 µm and 1 µmx1 µm scans. Green square represent area scanned at higher resolution.

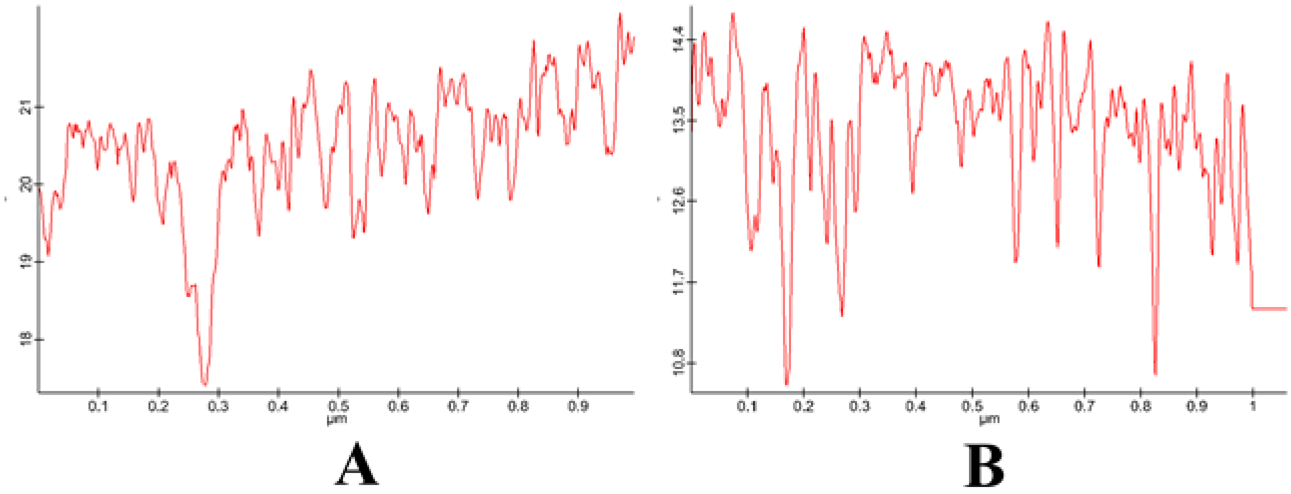

Figure S3. AFM surface phase scan crossection for nickel (II) alginate – A and cobalt (II) alginate – B.

Table S1. Gravimetric analysis of calcinated nickel (II) alginate. Samples were heated at 110°C for 1 h and weighted using an analytical balance. Values were calculated by subtracting the crucible's mass. Measurements were done in triplicate. Theoretical mass refers to mass of sodium alginate.

	Mass 1 [mg]	Mass 2 [mg]	Mass 3 [mg]	Average [mg]	Theoretical mass [mg]
Ni^{2+} alginate	17.32	16.97	17.03	17.10 +/- 0.18	18

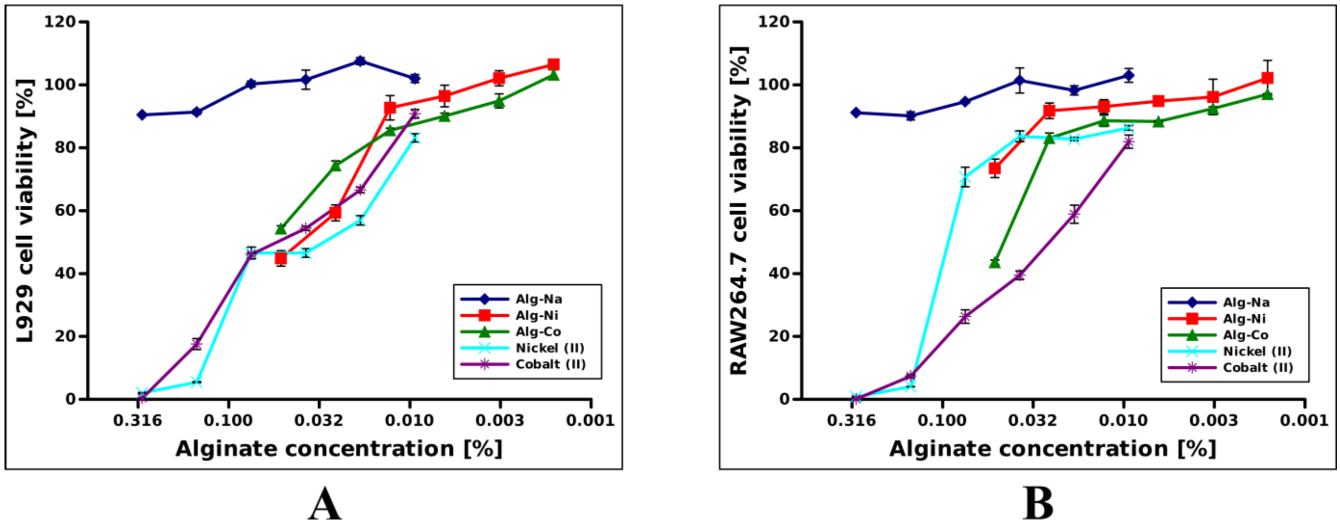

Figure S4. Cytotoxicity evaluation of nickel (II) and cobalt (II) alginates on different cell lines. A – L929 cell viability; B – RAW264.7 cell viability. Cell viability values were normalized considering positive control (cells cultured in DMEM alone) as having 100% viability. All samples were in triplicate. Values are represented as mean +/- SEM. X axis is log transformed.

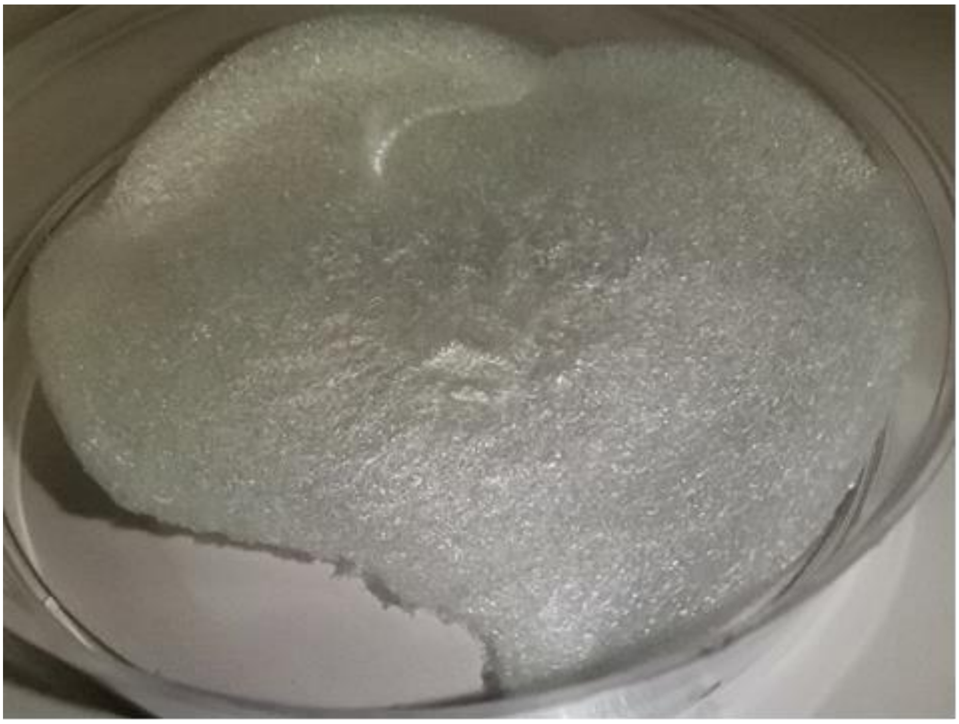

Figure S5. Lyophilized nickel (II) alginate sample with immobilized EGFP-HisTAG.
